# Molecular characterization of hepatitis B virus in liver disease patients and asymptomatic carriers of the virus in Sudan

**DOI:** 10.1186/1471-2334-13-328

**Published:** 2013-07-18

**Authors:** Mukhlid Yousif, Hatim Mudawi, Sahar Bakhiet, Dieter Glebe, Anna Kramvis

**Affiliations:** 1 Hepatitis Virus Diversity Research Programme, Department of Internal Medicine, Faculty of Health Sciences, University of Witwatersrand, 7 York Road, Parktown, Johannesburg 2193, South Africa; 2 Department of Medicine, Faculty of Medicine, University of Khartoum, Khartoum, Sudan; 3 Institute of Endemic Diseases, University of Khartoum, Khartoum, Sudan; 4 Institute of Medical Virology, National Reference Centre of Hepatitis B and D, Justus Liebig-University of Giessen, Giessen, Germany

**Keywords:** Bioinformatics, Genotype, Serotype, Sudan, Subgenotype, Africa, Phylogenetics

## Abstract

**Background:**

Hepatitis B virus is hyperendemic in Sudan. Our aim was to molecularly characterize hepatitis B virus from Sudanese individuals, with and without liver disease, because genotypes play an important role in clinical manifestation and treatment management.

**Methods:**

Ninety-nine patients - 30 asymptomatic, 42 cirrhotic, 15 with hepatocellular carcinoma, 7 with acute hepatitis and 5 with chronic hepatitis- were enrolled. Sequencing of surface and basic core promoter/precore regions and complete genome were performed.

**Results:**

The mean ± standard deviation, age was 45.7±14.8 years and the male to female ratio 77:22. The median (interquartile range) of hepatitis B virus DNA and alanine aminotransferase levels were 2.8 (2.2-4.2) log IU/ml and 30 (19–49) IU/L, respectively. Using three genotyping methods, 81/99 (82%) could be genotyped. Forty eight percent of the 99 patients were infected with genotype D and 24% with genotype E, 2% with putative D/E recombinants and 7% with genotype A. Patients infected with genotype E had higher frequency of hepatitis B e antigen-positivity and higher viral loads compared to patients infected with genotype D. Basic core promoter/precore region mutations, including the G1896A in 37% of HBeAg-negative individuals, could account for hepatitis B e antigen-negativity. Pre-S deletion mutants were found in genotypes D and E. Three isolates had the vaccine escape mutant sM133T.

**Conclusion:**

Sudanese hepatitis B virus carriers were mainly infected with genotypes D or E, with patients infected with genotype E having higher HBeAg-positivity and higher viral loads. This is the first study to molecularly characterize hepatitis B virus from liver disease patients in Sudan.

## Background

Hepatitis B virus (HBV), the prototype member of the family *Hepadnaviridae* is responsible for chronic infection of more than 240 million people worldwide [1], of which 65 million reside in Africa [2].

Sudan is an African country with high HBV seroprevalence of greater than 8% HBsAg-positivity, ranging from 6.8% in central Sudan to 26% in southern Sudan [3-5]. HBV infection occurs in early childhood in southern Sudan, with the infection increasing with age in northern Sudan [3,6]. HBV was shown to cause 22% of fulminant hepatitis cases in Sudan [7] and 18.5% of Sudanese blood donors were exposed to the virus and 4% were infected at the time of donation [8]. Nine genotypes of HBV, A–I, with a distinct geographic distribution have been recognized [9-11]. A tenth genotype, J, has been proposed but was found only in one person [12]. Genotype A, D and E circulate in Africa [2]. Genotype A prevails in southern, eastern and central Africa. Genotype D is the dominant genotype in northern Africa, whereas in western Africa genotype E predominates. Subgenotypes have also been identified within genotypes A and D [9,10].

Considering Sudan’s unique position and the flux of people across its borders, it is important that the HBV genotypes prevailing in this country are determined. In a single study, in Sudanese asymptomatic blood donors, it was found that 57.5% were infected with genotype E, 40.5% with genotype D and 2% with subgenotype A2 [8]. Moreover, a diversity of genotypes are distributed in neighbouring countries [2]: genotype D in Egypt to the north [13], genotype E in the Democratic Republic of Congo to the west [14] and genotype A in Kenya [15,16] and Uganda [17] to the south. Knowledge of the genotypes prevailing in Sudanese, with and without liver disease, is important in treatment management, as well as disease prognosis because genotypes play a role in both of these aspects [18,19].

The Regional committee for the World Health Organization (WHO) Eastern Mediterranean Region (EMR), to which Sudan belongs, urged member states to:

*“Improve the epidemiological surveillance systems, develop a hepatitis registry and implement serosurveys in order to produced reliable data to guide prevention and control measures and monitor impact of preventive strategies”*[20].

Thus our objective was to molecularly characterize HBV from HBsAg-positive persons with known clinical status.

## Methods

### Serum samples

A cross-sectional, laboratory based study was conducted. Ninety-nine sera were collected from HBsAg-positive patients referred to the hepatology and general medical clinics at IbnSina Hospital, Soba University Hospital and Khartoum Teaching Hospital in Khartoum State between August 2008 and March 2009. The clinical report forms (CRFs) were completed by qualified practitioners. Informed consent was obtained from each patient included in the study and the study protocol conforms to the ethical guidelines of the 1975 Declaration of Helsinki as reflected in *a priori* approval by the Human Ethics Committees of the University of the Witwatersrand and the University of Khartoum and the Ministry of Health of Sudan. Alanine amino transferase (ALT) levels were determined (reference range 5–40 IU/L) [21] and samples stored at -20^0^C. Chronic carriers were infected for longer than six months and were classified as asymptomatic carriers (ASCs) if they had normal ALT or chronic hepatitis (CH) patients if they had abnormal ALT. Acute hepatitis (AH) cases were diagnosed based on clinical presentation (symptoms and clinical presence of jaundice) plus high ALT and the presence of hepatitis B core IgM antibody (HBcAbIgM). Clinical and ultrasonographic evidence were used to diagnose cirrhotic (CR) and hepatocellular carcinoma (HCC) cases.

### HBV serology

HBsAg was assayed using Monolisa™ HBsAg ULTRA and HBeAg using Monolisa™ HBeAg-Ab PLUS kit (Bio-rad, Hercules, CA). Anti-HBe and anti-HBc antibodies were determined using HBeAg/Anti-HBe ELISA and Anti-HBc Total ELISA, respectively (DIAsource ImmunoAssays S.A, Nivelles, Belgium). Anti-HBc IgM was determined using the ARCHITECT® kit (Abbott Diagnostics, Wiesbaden, Germany).

### DNA extraction

DNA was extracted from 200 μL of serum using QIAamp DNA mini blood kit (QIAGEN GmbH, Germany), according to manufacturer’s instructions, and eluted in 100 μL of buffer.

### Real-time PCR quantification of HBV DNA

PCR primers, HBV-Taq1 and HBV-Taq2 covering a region of the S gene (321 to 401 from the *Eco*RI site) with a FAM/TAMRA labelled TaqMan BS-1 probe [22] were used to quantify HBV DNA in an ABI 7500 Real Time PCR System (Applied Biosystems, Foster City, Ca, USA). A serial dilution of cloned plasmid DNA containing a single genome of HBV DNA, with concentrations ranging from 2 × 10^1^ to 2 × 10^11^ IU/ ml, was used as template to generate the standard curve. The second WHO International Standard for HBV Nucleic Acid Amplification Techniques (product code 97/750 National Institute for Biological Standards and Controls (NIBSC); Hertfordshire, UK), which has a final concentration of 10^6^ IU/ml was used as the internal standard. The standard curve, blank, positive and negative controls, and samples were all tested in duplicate. The measured IU/ml for each reaction was calculated using the Ct (cycle threshold) value of each PCR interpolated against the linear regression of the standard curve. The lower detection limit of our assay is ~20 IU/ ml. The conversion formula of IU =copies/4.7 was used [23-25].

### Polymerase chain reaction (PCR) and restriction fragment polymorphism assay (RFLP)

The basic core promoter/precore (BCP/PC) region and complete S open reading frame (ORF) were amplified in a MyCycler™ thermocycler (Bio-Rad, Hercules, Ca, USA) using Promega Taq DNA polymerase (Promega, Madison, WI). The BCP/PC PCR was amplified using a slight modification of the method described by Takahashi and colleagues [26] primers 1606 (+) (1606–1625 from *Eco*RI site) and 1974 (−) (1974–1955 from *Eco*RI site) were used for the first round (denaturation 94°C for 1 min, annealing 55°C for 1 min, extension 72°C for 2 min, 40 cycles) and 1653(+) (1653–1672 from *Eco*RI site) and 1959(−) (1959–1940 from *Eco*RI site) for the second round, with the identical cycling conditions as the first-round PCR [25,27]. A nested PCR was carried out to amplify the complete S ORF : primers 2410(+) (2410–2439 from *Eco*RI site) and 1314(−) (1314–1291 from *Eco*RI site) were used for the first round (denaturation 94°C for 1 min, annealing 66°C for 1 min, extension 72°C for 3 min, 40 cycles) and 2451 (+) (2451–2482 from *Eco*RI site) and 1280 (−) (1280–1254 from *Eco*RI site) for the second round (denaturation 94°C for 1 min, annealing 65°C for 1 min, extension 72°C for 3 min, 40 cycles) [27]. Another nested PCR reaction was carried out to amplify a short region of the S (459–710 from *Eco*RI site) to determine the genotype: primers 255 (+) (253–275 from *Eco*RI site) and 759 (−) (759–739 from *Eco*RI site) were used for the first round (denaturation 94°C for 30 sec, annealing 56°C for 40 sec, extension 72°C for 1 min, 40 cycles) and [459(+) (460–480 from *Eco*RI site)] and [710(−) (711–691 from *Eco*RI site)] for the second round (denaturation 94°C for 30 sec, annealing 56°C for 40 sec, extension 72°C for 50 sec, 40 cycles) [28]. When the complete S ORF and the short S could not be amplified, a RFLP assay was used to determine the genotype of the HBV isolates. Primers P7 (256–278 from *Eco*RI site) and P8 (796–776 from *Eco*RI site) were used to amplify nucleotides 256–796 of the S region. The amplicon was then cleaved using restriction enzymes *Hin*fI and *Tsp*509I, in separate reactions, to give the characteristic RFLP patterns for the different genotypes [29]. The complete genome was amplified using a single amplification method with primers P1 (1821–1841 from *Eco*RI site) and P2 (1825–1806 from *Eco*RI site), with modifications in the cycling conditions [30]. Initial denaturation at 98°C for 1 min, 80°C for 30 sec followed by adding the polymerase mix, 98°C for 1 min, then 35 cycles of: 98°C for 10 sec, 57°C for 30 sec and 72°C for 1 min.

### Sequencing

The BigDye Terminator v3.0 Cycle Sequencing Ready Reaction Kit (Applied Biosystems., Foster City, USA) was used and sequencing performed using the ABI 3130XL Genetic analyzer (Applied Biosystems, Foster City, CA). In addition to sequencing primers: 2497F (2497–2516), 3188F (3188–3206) and 591F (591–611) used previously [27,31], four new primers: 1069F (1069–1088) (5′- TGT ATT CAA TCT AAG CAG GC-3′), *Post-Bgl* III (2000–2017) (5′-CCG ATA CAG AGC TGA GGC-3′), 2022F (2022–2041) (5′-CCT TAG AGT CTC CTG AGC AT-3′), 2480R (2461–2480) (5′-CAC CTT ATG AGT CCA AGG AA-3′), were used to sequence the complete genome. All positions are numbered from the *Eco*RI site of genotype A (AY233286). The three overlapping fragments of the complete S, and the 7 fragments of the complete genome thus obtained, were assembled using the Fragment Merger Tool [32]. Sequences were deposited in GenBank, accession numbers KF170739-KF170812.

### Phylogenetic analysis

Neighbour-joining using MEGA5 [33] or PHYLIP (Phylogeny inference package version 3.69) or DNADIST consecutively with NEIGHBOR were used to generate dendrograms [34].

### Analysis for intergenotypic recombination

SimPlot 3.5.1©, an interactive 32-bit software program, that plots distances (or similarity) versus position [35] was used to show recombination in isolate SDAC031 [33].

## Results

### HBV serology and viral loads

Of the 99 patients, 77 were males and 22 were females. Mean age ± standard deviation (SD) was [45.7±14.8] years. All sera were HBsAg- and anti-HBc-positive; 12 were HBeAg-positive/anti-HBe-negative, 75 were HBeAg-negative/anti-HBe-positive, and 12 had neither HBeAg nor anti-HBe. All patients were HBV DNA-positive, with a median (interquartile range) (IQR) viral load of [2.8 (2.2–4.2)] log IU/ml and ALT median (IQR) level was [30 (19–49)] IU/L (reference range 5–40 IU/L) [21].

### Clinical and demographic characteristics

The 99 cases were classified into five clinical groups: HCC (n=15), CR ( n=42), ASCs (n=30), AH ( n=7) and CH ( n=5). The HCC patients were significantly older than patients in the other groups (p <0.05) and the AH patients were significantly younger (p <0.05). The ALT levels were the highest in the AH (p <0.05) (Table 1). The median viral load of HBeAg-positive patients [6.99 (5.63–7.57) log IU/ml] was significantly higher than in HBeAg-negative patients [2.55 (2.09–3.42) log IU/ml] (p<0.05). The viral loads and frequency of HBeAg-positivity did not differ between clinical groups.

**Table 1:** Demographic and clinical characteristics of 99 patients

	**Clinical status**				
	**HCC (15)**	**ASC (30)**	**CR (42)**	**AH (7)**	**CH (5)**
Male: Female	13:02	22:08	37:05:00	02:05	03:02
Age mean ±SD	57.3±13.8^a^	42.8±15.1	46.5±12.9	32.1±12.4^a^	37.6±41.0
ALT median IU/L (IQR)	40 (24–50)	22 (18–33)	27 (18–45)	461 (312–834)^a^	38 (29–47)
HBeAg positive (%)	2 (13.3)	2 (6.7)	7 (16.7)	1 (14.3)	0
Log viral loads	3.8	2.5	2.8	3	2.1
IU/ml median (IQR)	(2.4-4.7)	(2.2–3.3)	(2.2-5.3)	(2.2-3.8)	(2.0-3.6)

*HCC* Hepatocellular carcinoma, *ASC* Asymptomatic carriers, *CR* Cirrhosis, *AH* Acute hepatitis, *CH* Chronic hepatitis.

^a^Significant difference with other groups.

### HBV genotyping and phylogenetic analysis

**Figure 1:**
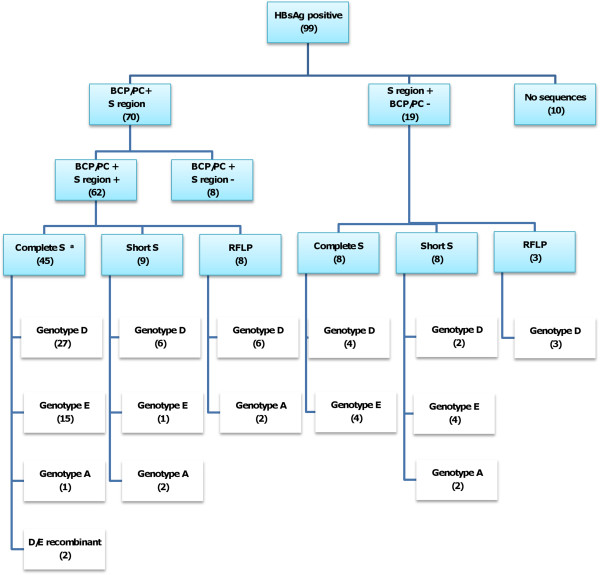
**Algorithm summarizing protocol and results of the molecular characterization of BCP/PC region and genotyping of HBV. **^a^ Three isolates showed genotype D based on S region and genotype A based on BCP/PC region.

In order to maximize the number of isolates genotyped, three methods were used sequencially (Figure 1).

81 HBV isolates were thus successfully genotyped

**Figure 2:**
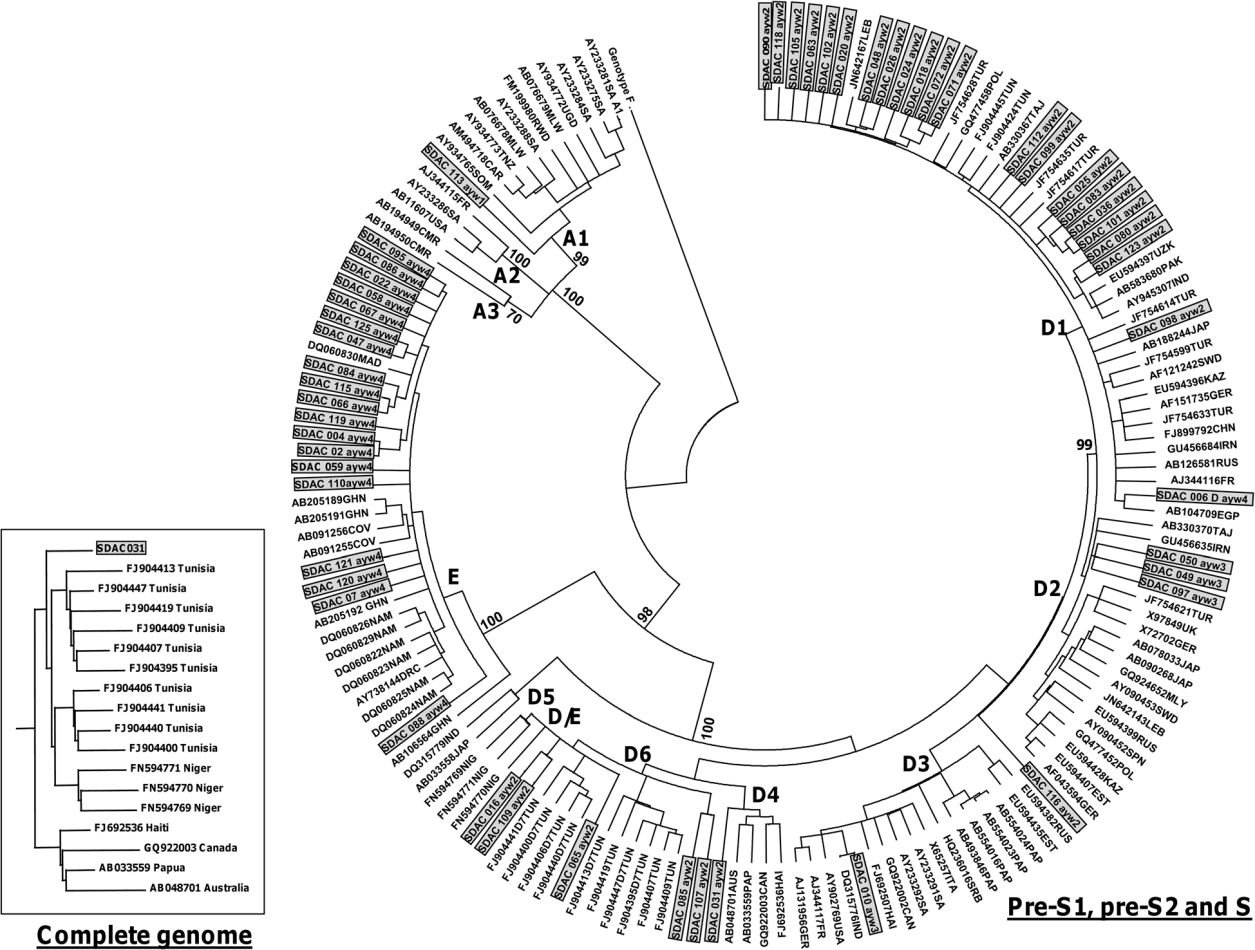
**A rooted phylogenetic tree of 53 complete S sequences of HBV obtained from Sudanese liver disease patients (shaded in box) with 104 reference HBV sequences, using neighbour-joining.** Bootstrap statistical analysis was performed using 1000 datasets, indicated as percentages on the nodes. The sequences are labeled by their accession numbers and country. [AUS, Australia; CMR, Cameron; CAN, Canada; CAR, Central African Republic; CHN, China; COV, Côte d’Ivoire; DRC, Democratic Republic of Congo; EGP, Egypt; EST, Estonia; FR, France; GHN, Ghana; GER, Germany; HAI, Haiti; IND, India; IRN, Iran; ITA, Italia; JAP, Japan; KAZ, Kazakhstan; LEB, Lebanon; MAD, Madagascar; MLY, Malaysia; MLW, Malawi; NAM, Namibia; NIG, Niger; PAK, Pakistan; PAP, Papua New Guinea/Indonesia; POL, Poland; RUS, Russia; RWD, Rwanda; SRB, Serbia; SOM, Somalia; SA, South Africa; SPN, Spain; SDW, Sweden; TNZ, Tanzania; TAJ, Tajikistan; TUN, Tunisia; TUR, Turkey; UGN, Uganda; UK, United Kingdom; USA, United States; UZK, Uzbekistan ]. The letters, A, D and E, represent the genotypes and the numbers the subgenotypes. A rooted phylogenetic tree of the complete genome of SDAC 031 (shaded) relative to 17 reference HBV sequences, using neighbour-joining is shown in the left hand box. When the complete genome was analyzed SDAC031 clustered with D6 whereas when the S region alone was analyzed it clustered with D4.

Firstly, 53 of 99 were genotyped using amplification and sequencing of the complete S (2848–835 from *Eco*RI), followed by phylogenetic analysis (Figure 2), secondly,17 of 46 were genotyped by amplification and sequencing of a short S fragment (520–704 from *Eco*RI), followed by phylogenetic analysis (trees not shown) and thirdly,11 of 29 by RFLP analysis.

The relatively longer amplicon of the S region of 2.1 kb in length, compared to the shorter regions used for the short S and RFLP amplifications, meant that not all samples could be amplified in the longer region successfully. 18.2% (18/99) could not be genotyped using any of the three methods. No sequences could be obtained for 10 of these isolates and for the remaining 8 only the BCP/PC region was sequenced, which is not sufficient to differentiate between genotypes D and E.

The genotype distribution in the 81 samples was 59% genotype D: 30% genotype E: 8.5% genotype A: 2.5% putative D/E recombinant. If we include the 18 samples that could not be genotyped, 48.5% (48/99) were infected with genotype D, 24.2% (24/99) with genotype E, 7.1% (7/99) with genotype A and 2% (2/99) were infected with a putative D/E recombinant.

Phylogenetic analysis of the complete S region allowed for further classification into subgenotypes (Figure 2). Based on the recently suggested classification system [33], 23 of 31 genotype D isolates belonged to subgenotype D1 (74%), 3 to D2 (10%), 1 to D3 (3%) and 4 to D6 (13%). One genotype A isolate belonged to subgenotype A1 (Figure 2).

**Figure 3:**
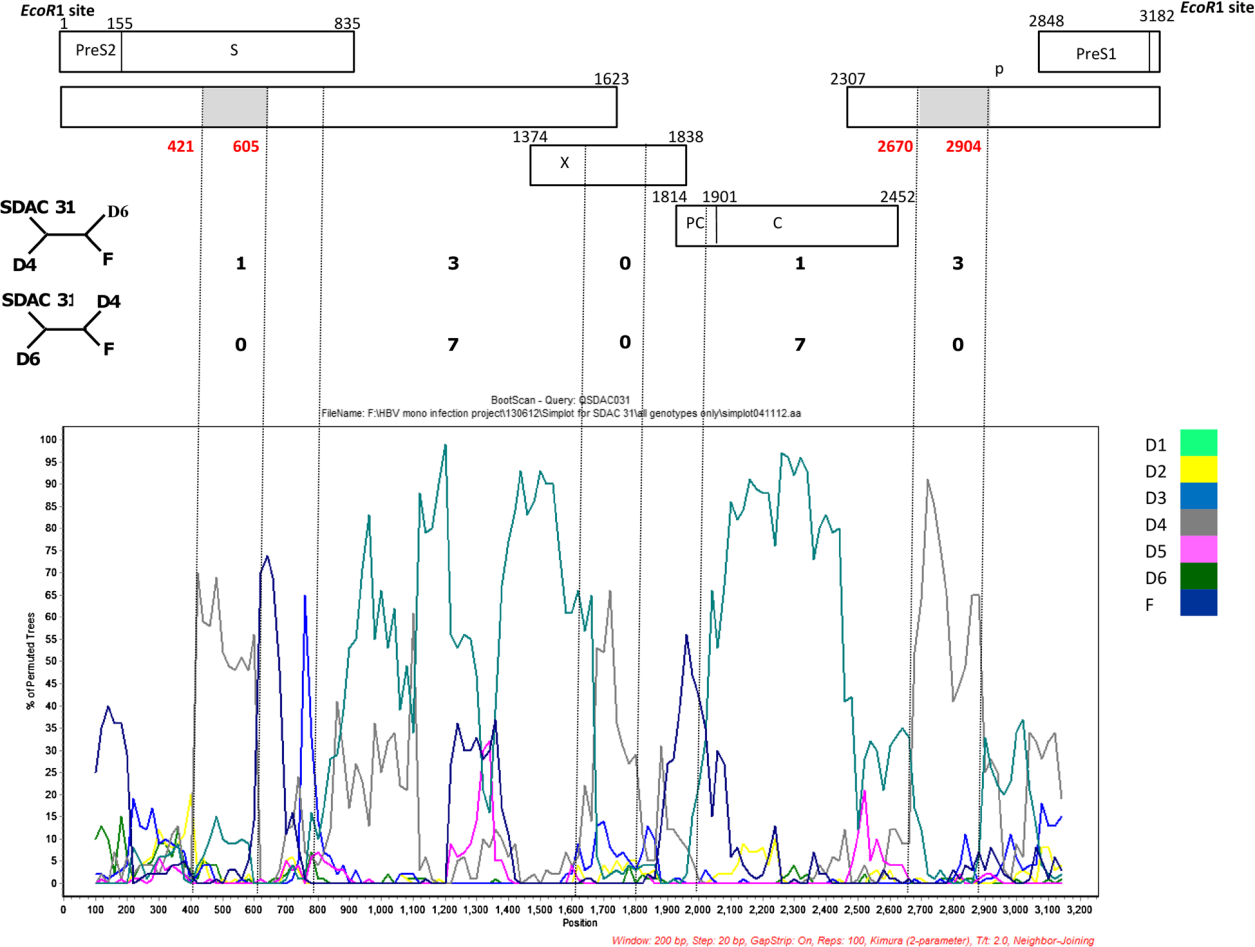
**Graphs show bootscanning values of query sequence SDAC031 to representative sequences of subgenotypes D1 to D6 and outgroup, genotype F.** Graphs were generated using Simplot version 3.5.1^©^ with window size: 200 bp, step size 20 bp, gap-strip off, 100 bootstrap replicates, Kimura transition/transversion ratio:2 and NEIGHBOR. Dashed vertical lines indicate breakpoints. The numbers of informative sites, which are shared by the isolate with subgenotype D4 are shown above and with subgenotype D6, below, on the right hand side of the four-member trees representing these sites. The genomic regions belonging to subgenotype D4 are shaded in *grey*.

The complete genome of four isolates was amplified, two (SDAC024 and SDAC031) belonged to genotype D and two (SDAC047 and SDAC125) to genotype E. Although the genotypes determined using the complete genome agreed with that determined using the S ORF, there was a discrepancy in the subgenotype classification of SDAC031. Using phylogenetic analysis of the complete S, it clustered as an outlier of subgenotype D4, whereas following complete genome phylogenetic analysis it clustered with D6 (Figure 2). Simplot analysis showed SDAC031 to be a recombinant of D6 and D4. By mapping the informative sites, we estimated the transition positions between D6 and D4 (Figure 3). The genotype E isolates (SDAC047 and SDAC125) clustered together when the complete S was compared, whereas they separated into different clades in the complete genome analysis (*data not shown*).

Although the majority of subgenotype D isolates showed the respective distinct subgenotype amino acids signatures [33], there were exceptions. SDAC031 (D6) showed preS2I42T, spV59F and rtT237P. SDAC085 (D6) had spV59S. SDAC107 (D6) had preS2I42T and rtT237P. All 17 genotype E isolates had genotype E signature amino acid motifs.

### Comparison of patients infected with genotypes D and E

Because the majority were infected with either genotype D or E, further analyses compared the patients’ clinical and virological features infected with these genotypes. No significant difference was observed in gender, age, ALT and clinical groups of patients infected with either genotype. Patients infected with genotype E, showed a significantly higher frequency of HBeAg-positivity compared to patients infected with genotype D (p <0.05) and HBV DNA levels in patients infected with genotype E were significantly higher (p <0.05) (Table 2).

**Table 2:** Clinical and demographic characteristics of 81 patients, for which HBV was genotyped

	**Clinical status**					**Demographic**		**Biochemical and virological**		
						**characteristics**		**characteristics**		
	**HCC (13)**	**ASC (24)**	**CR (33)**	**AH (7)**	**CH (4)**	**Male: Female**	**Age mean ±SD**	**ALT median IU/L (IQR)**	**HBeAg positive (%)**	**Viral loads log median IU/ml (IQR)**
Genotype D (%)	4(30.7)	14(58.3)	23(69.7)	4(57.1)	3(75)	38:10	44.6 ±15.1	29 (19–57)	4 (8.3)	2.8 (2.0-3.9)
Genotype E (%)	6(46.2)	6(25)	8(24.3)	3(42.9)	1(25)	17:07	49.3 ±14.3	29 (18–65)	7 (29.2)^a^	4 (2.6-5.7)^a^
Genotype D/E (%)	1(7.7)	0	1(3)	0	0	2:0	56.5 ±7.0	NA	0	2.4 (2.3-2.41)
Genotype A (%)	2(15.4)	4(16.7)	1(3)	0	0	5:2	38.14 ±14.51	33 (28–54)	1 (14.3)	2.6 (2.3-3.9)

*HCC*, Hepatocellular carcinoma; *ASC*, Asymptomatic carriers; *CR*, Cirrhosis; *AH*, Acute hepatitis, *CH*, Chronic hepatitis.

^a^Significant difference between patients infected with genotype D or E.

### Molecular characterization of HBV isolates

#### Analysis of basic core promoter/precore (BCP/PC) region

**Figure 4:**
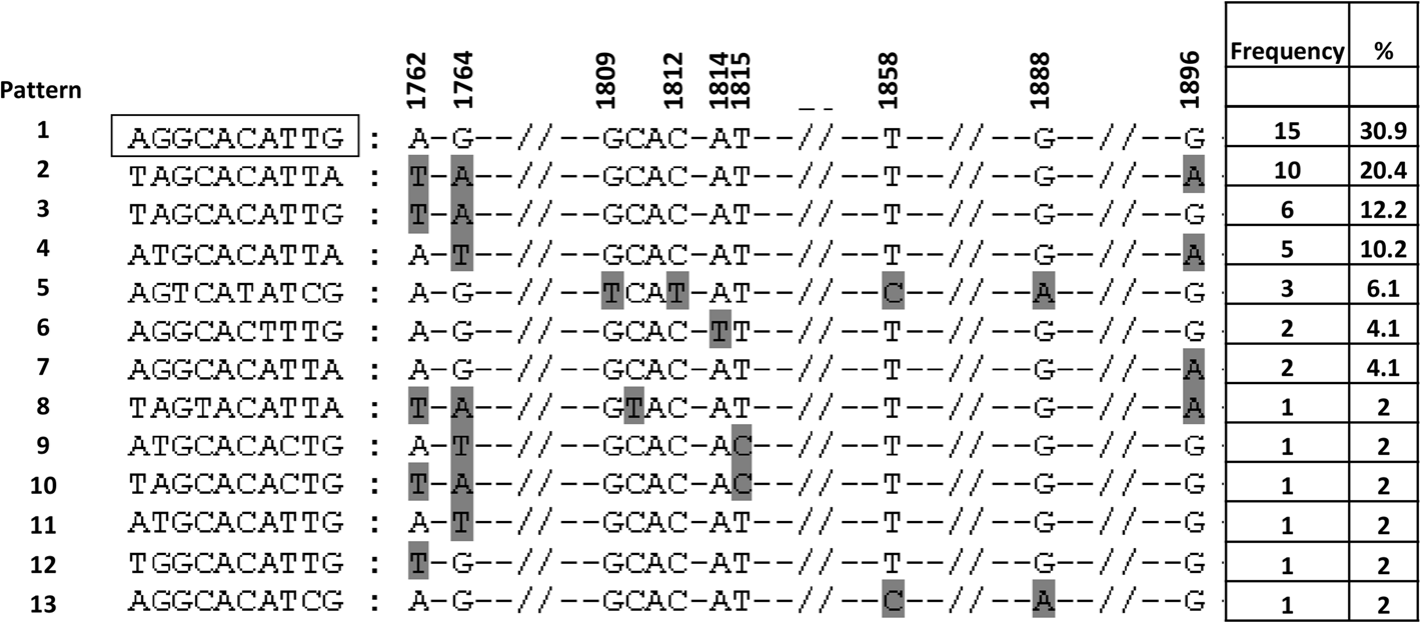
**Mutation distribution in the basic core promoter/precore region, at 11 loci of interest (1762, 1764, 1809–1812, 1814, 1815, 1858, 1888 and 1896), of 49 genotype D or E HBV isolates from HBeAg-negative patients.** Boxed motif represents the wild- type of genotypes D or E. Data were obtained using the Mutation Reporter Tool [36]*.* Mutations are shaded in *grey*. The frequency of the mutational patterns did not differ between genotype D and E.

Of the 70 isolates amplified and sequenced in the BCP/PC region, 62 amplfied in the S region and thus had genotype assignments. 57 isolates belonged to either genotype D or E, and 5 to genotype A. Seven of 8 genotype D or E isolates from HBeAg-positive patients had wild-type BCP/PC, and one (SDAC118) had G1764T. The mutations found in genotype D or E isolates from HBeAg-negative individuals are shown in Figure 4: 15/49 genotype D or E isolates from HBeAg-negative sera, had wild-type BCP/PC region. In 22/49 HBeAg-negative sera, the absence of HBeAg was as a result of missense mutations affecting the translation of the HBeAg precursor: 18 had the classical stop codon mutation G1896A (p< 0.05, when comparing isolates from HBeAg-negative and –positive sera) and 4 had initiation codon mutations, 2 A1814T and 2 T1815C. A1762T/G1764A was found in 11 G1896A mutants and in one T1815C mutant. Six isolates from HBeAg-negative sera had A1762T/G1764A alone. The Kozak sequence was GCAC at 1809–1812 in 45 isolates, GTAC in isolate SDAC 121 and TCAT with 1858C and 1888A in isolates SDAC018, SDAC090 and SDAC091. SDAC050 had 1858C with 1888A alone.

Although generally the BCP/PC region cannot be used to determine specific genotypes, genotype A and its subgenotypes can be differentiated from genotypes D or E because of specific sequence characteristics. One genotype A isolate from a HBeAg-positive patient, (SDAC108) had A1762T/G1764A. From the BCP/PC sequences it was deduced that this isolate belonged to subgenotype A2, because it had GCAC at 1809–1812, 1858C and 1888G. The other four genotype A isolates were from HBeAg-negative individuals. These isolates, SDAC062, SDAC073 SDAC100 and SDAC113, belonged to subgenotype A1 because they had TCAT at 1809–1812, 1858C and 1888A. One isolate had wild-type BCP/PC region, two had A1762T/G1764A, one with G1862T and the fourth isolate had G1862T alone. No significant association was found between the BCP/PC mutations and genotypes or clinical groups.

#### Analysis of the pre-S1, pre-S2, Surface and Polymerase regions

The complete S region was sequenced for 53 isolates and pre-S mutations were detected in 14 (26%). Four mutational patterns were identified: pre-S1 deletions (2/14; 14%), pre-S2 start codon mutation (4/14; 29%), pre-S2 start codon mutation with pre-S2 deletion (1/14; 7%) and pre-S2 deletion alone (7/14; 50%) (Table 3). Transcription regulatory box domain substitutions [CCAAT→CTAAT] (803–807 from *Eco*RI site), were found in two isolates (SDAC095 genotype E and SDAC098 genotype D). Three *cis-*acting elements mutations were found: C1092T in the Enhancer I in one isolate, C1155T in the Enhancer I in 6 isolates and T3109G S2 promoter in 2 isolates. PreS2F22L was found in 6 genotype E and three genotype D isolates. The vaccine escape mutation, sM133T, was observed in genotype E isolates, SDAC022, SDAC067 and SDAC088. In the reverse transcriptase domain of the polymerase, the following mutations were detected: rtA194T in SDAC049 (genotype D), rtV207M in SDAC020 (genotype D), rtS213T in SDAC063 and SDAC098 (genotype D), SDAC002, SDAC121 and SDAC004 (genotype E), rtV214A in SDAC059 (genotype E) and rtS215Q in SDAC049 and SDAC090 (genotype D).

**Table 3:** Pre-S region mutations and deletions

	**Clinical group**	**Genotype**	**Pre-S2 S tart codon**	**Deletions**					
				**Region**	**Nucleotides**		**Amino acids**		**Region and functions affected**
**ID**					**Size**	**Position**	**Size**	**position**	
SDAC024	CR	D	ATG	Pre-S2	21	35-55^a^	7	16-22^a^	
SDAC048	CR	D	ATG	Pre-S1	72	2998-3069	24	51-74	S promoter, Transactivator domain
SDAC083	CR	D	ATG	Pre-S2	21	35-55	7	16-22	
SDAC097	CR	D	*GTG*^b^	Pre-S 2	9	45- 53	3	19-21	
SDAC098	CH	D	ATG	Pre-S1	18	3129-3146	6	94-99	Hsc70 binding site, CCAAT binding factor (CBF) binding site, Cytosolic anchorage determinant (CAD)
SDAC112	ASC	D	ATG	Pre-S2	6	49-54	2	20-21	
SDAC118	CR	D	ATG	Pre-S2	54	4-57	18	5 to 22	pHSA binding site, Viral secretion
SDAC107	CR	D	*ATA*	-	-	-	-	-	-
SDAC090	CR	D	*ATA*	-	-	-	-	-	-
SDAC058	CR	E	ATG	Pre-S2	9	58-66^c^	3	20-22^c^	Transactivator domain
SDAC059	HCC	E	*GTG*	Pre-S2	12	52-63	4	18-21	Transactivator domain
SDAC088	HCC	E	ATG	Pre-S2	12	52-63	4	18-21	Transactivator domain
SDAC095	HCC	E	ATG	Pre-S2	15	52-66	5	18-22	Transactivator domain
SDAC067	ASC	E	*GTG*	-	-	-	-	-	-
SDAC113	ASC	A	*ACA*	-	-	-	-	-	-

^a^ nucleotide or amino acid position relative to genotype D (GU456684).

^b^ mutant pre-S2 start codon *italicized.*

^c^ nucleotide or amino acid position relative to genotype E (AB205191).

## Discussion

In Sudan, where HBV is hyperendemic, little is known about the genotypes and mutants of HBV in liver disease patients. Thus HBV, from 99 patients, belonging to five clinical groups, were studied. Of the 81 samples that were successfully genotyped, close to 60% belonged to genotype D whereas the opposite was true in Sudanese blood donors, where genotype E prevailed (57%) [8]. HCC patients in this study, who were infected with either genotype D or E, were significantly older than ASC, CH, AH, CR patients, with the AH patients being significantly younger (Table 1). This differs from HCC patients, infected with genotype A, who develop cancer at a significantly younger age [28]. In agreement with others [37,38], the HCC group had the highest median viral load. High viral loads have been implicated as a risk factor for HCC development [39].

In agreement with the study in Sudanese blood donors [8], the predominant subgenotype of D was D1. Furthermore, following phylogenetic analysis of the complete S region, two isolates, which lacked the 33 nucleotide deletion, characteristic of genotype D, were genotype D/E recombinants [8,33,40]. Following phylogenetic analysis of the complete S, one isolate belonged to subgenotype A1, and from the BCP/PC sequence, a further four were deduced to belong to subgenotype A1 [41]. This is the first time that subgenotype A1 has been identified in Sudan whereas a previously sequenced genotype A isolate from Sudan belonged to A2 [8]. Subgenotype A1 circulates in southern and eastern Africa and Southern Asia and A2 is found in Northern and Central Europe and North America [42].

Discrepant results were obtained in the subgenotype assignment of SDAC031 when the complete S and complete genome were analyzed. The complete S clustered as an outlier of D4 whereas the complete genome clustered with D6. D4 and D6 are phylogenetically closely related (Figure 2). Further analysis using Simplot, showed that it was a D6/D4 recombinant (Figure 3) and the majority of signature amino acids were of D6, with the exception of a number of amino acids, which were of D4 [33]. Geographically, D6 is distributed in theMaghreb and Madagascar, whereas D4 in the Americas and Australia. It is possible that D4 originated in Africa but has subsequently been replaced by other subgenotypes of D and the recombinant is a remnant of the original strain/s.

The HBV genotypes show a distinct geographical distribution in Africa, with genotype D predominating in the North, genotype E in the West and genotype A in the South-East [9]. This is the first study to describe the co-circulation of genotypes D and E in liver disease patients and to allow the comparison of patients infected with these two genotypes. In agreement with a study carried out in Sudanese blood donors [8], viral loads were significantly higher in genotype E-infected patients compared to genotype D-infected, with patients infected with genotype E, showed a significantly higher frequency of HBeAg-positivity. The small size of HBeAg allows it to traverse the placenta and elicit HBe/HBcAg-specific T helper cell tolerance *in utero*[43]. Thus babies born to HBeAg-positive mothers have high chronicity rates than those born to HBeAg-negative mothers [43]. The high frequency of HBeAg-positivity in mothers infected with genotype E, would lead to its vertical transmission and explain the high prevalence and geographical restriction of this genotype in Africa and to African emigrants to other regions [14]. The higher HBeAg-positivity seen in individuals infected with genotype E, could confer tolerance and less serious clinical manifestations than genotype D, where HBeAg-positivity was lower. This could explain why genotype E prevails in the Sudanese blood donors [8], whereas genotype D prevailed in the liver disease patients in the present study.

The majority of the HBeAg-negativity was as a result of the classical G1896A, which abolishes HBeAg expression [44] and occurs in genotype D or E but not A because the encapsidation signal secondary structure precludes this mutation in genotype A [45,46]. Other mutations including transcriptional A1762T/G1764A and translation initiation mutations were responsible of HBeAg-negativity in a number of patients. Three isolates had TCAT instead of GCAC in the Kozak sequence preceding the precore initation codon and can affect HBeAg expression at the translational level [47]. This Kozak mutation occurred together with 1858C and 1888A (pattern 5, Figure 4), which are characteristics of subgenotype A1 [41]. One isolate has 1858C and 1888A alone. However, following phylogenetic analysis of the complete S region, these four isolates were found to belong to genotype D. It is possible that these patients were co-infected with genotypes D and A or with D/A recombinants. These possibilities can only be discriminated by complete genome cloning and sequencing.

Four different pre-S mutational patterns were identified (Table 3). In genotype E, pre-S2 deletions were found mainly in HCC patients, whereas in genotype D, the deletion mutants were from non-HCC patients. However, the numbers were small to reach any firm conclusions. Pre-S deletion mutants were found in genotype E isolates from ASCs from Guinea [48] and Sudan [8]. Pre-S deletion/mutations affect the progression to serious liver disease in patients infected with either genotype B or C [49].

Six genotype E and three genotype D isolates had preS2F22L, which is a risk factor for HCC [50,51]. Interestingly, three genotype E isolates had the sM133T mutation, which could possibly compromise antibody neutralization and may represent potential vaccine escape mutants [52]. However, because these individuals were not vaccinated for HBV, this mutation may have emerged as a result of host immune pressure. The reverse transcriptase mutations rtA194T, rtV207M, rtS213T, rtV214A and rtS215Q detected in the present study are neither primary resistance mutations, nor have they ever been seen in overt resistance during therapy [53]. When mutations rtA194T, rtV207M rtS213T rtV214A and rtQ215S were tested in our sensitive and reliable *in vitro* resistance test system, the mutants showed no resistance to lamivudine (LMV), entecavir (ETV), adenofovir (ADF) and tenofovir (TDF). (Glebe *et al.*, unpublished observations).

## Conclusion

This is the first study to molecularly characterize HBV from Sudanese liver disease patients, who were predominantly infected with genotypes D and E, allowing comparison of the effect of these genotypes on clinical manifestation in the same ethnic group.

## Abbreviations

AH: Acute hepatitis; ALT: Alanine amino transferase; ASC: Asymptomatic carriers; BCP/PC: Basic core promoter/precore; CH: Chronic hepatitis; CRF: clinical report form; DFG: Deutsche Forschungsgemeinschaft (German Research Foundation); DNA: Deoxyribonuclease; HBcAb: Hepatitis B core antibody; HBeAb: Hepatitis B e antibody; HBeAg: Hepatitis B e antigen; HBsAb: hepatitis B surface antibody; HBsAg: Hepatitis B surface antigen; HBV: hepatitis B virus; HCC: Hepatocellular carcinoma; IQR: Interquartile range; CR: cirrhosis; ORF: open reading frame; PCR: Polymerase chain reaction; RFLP: Restriction fragment polymorphism; rt: Reverse transcriptase; s: Surface; sp: Spacer; WHO: World Health Organization.

## Competing interests

The authors declare that there are no competing interests.

## Authors’ contributions

Conceived the study: AK, DG, HM; Designed the experiments: MY, DG, AK; Performed the experiments:MY; Analyzed the data: MY, AK; Contributed reagents/materials: AK, SB, HM; Wrote the paper: MY, AK Read, contributed to and approved the paper: MY, HM, SB, DG, AK. All authors read and approved the final manuscript.

## Pre-publication history

The pre-publication history for this paper can be accessed here:

http://www.biomedcentral.com/1471-2334/13/328/prepub
